# Microbe-Induced Abiotic Stress Alleviation in Plants: From Hidden Partners to Central Drivers of Resilience

**DOI:** 10.3390/plants15101427

**Published:** 2026-05-07

**Authors:** Ying Ma

**Affiliations:** Centre for Functional Ecology, Associate Laboratory TERRA, Department of Life Sciences, University of Coimbra, Calçada Martim de Freitas, 3000-456 Coimbra, Portugal; cathymaying@gmail.com

## 1. Introduction

Our understanding of plant stress biology has shifted with debate toward a more integrative, microbiome-centered perspective. What was once primarily viewed as an intrinsic, plant-centered physiological problem is now often interpreted as a system-level outcome shaped by the interactions between plants and their associated microbiomes. Microorganisms, long considered background inhabitants of soil and plant tissues, are now thought to play a more decisive role in determining plant fitness under environmental stress.

Abiotic stress imposes one of the most severe constraints on global food production and ecosystem stability [1]. Drought, salinity, extreme temperatures, and heavy metal contamination together cause a large proportion of crop yield losses worldwide and are likely to intensify under climate change [2]. However, plants do not face these stresses alone. Evidence accumulated since the mid-2010s suggests that rhizosphere microbes, endophytes, and phyllosphere communities strongly influence the responses of plants to stress by modulating nutrient acquisition, hormone signaling, redox balance, and ion transport [3]. Microbial functions such as phosphate solubilization, nitrogen fixation, exopolysaccharide (EPS) production, siderophore release, and metal immobilization or volatilization are frequently linked to increases in the tolerance of plants to stress [3].

Recent studies have increasingly emphasized the importance of microbial contributions; however, this perspective is not fully supported, especially when moving from controlled environments to field conditions [4]. As climate models now integrate biosphere–atmosphere feedbacks, modern plant stress biology must incorporate the microbiome as a coregulator of plant adaptation [5]. Despite this recognition, our conceptual and experimental frameworks do not fully capture the ecological reality in which plants experience multiple, interacting stresses in microbially complex environments.

The overall picture, however, hides important mechanistic and institutional blind spots. Much of what we know about microbe-mediated stress alleviation has been derived from simplified laboratory systems using single microbial strains and single stress factors [3]. These approaches have been invaluable in identifying the beneficial traits of microbes, but they inaccurately represent real agricultural soils, where plants simultaneously encounter fluctuating water availability, salinity, nutrient limitation, and toxic metal conditions while interacting with thousands of microbial taxa [6]. As such, the performance of microbial inoculants identified through these approaches has been inconsistent in the field, underscoring the need to transition from strain-based to community-based and system-level perspectives.

Plants are not passive in this process. Under stress, plants can actively reshape their associated microbiomes through changes in root exudation and immune signaling. Stress-induced metabolites and signaling compounds may selectively enrich the abundance of microbes capable of buffering osmotic stress, detoxifying harmful elements, or enhancing antioxidant defenses [7]. However, the mechanisms underlying these recruitment processes remain only partly understood. In particular, the direct links between microbial genes, metabolites, and plant stress response pathways have been difficult to establish.

Stress is also highly heterogeneous in space, although this aspect has often been overlooked in research. Soil structure, moisture, redox conditions, and nutrient gradients create microhabitats that strongly influence microbial activity [8]. In saline or heavy-metal-contaminated soils, microbial EPS and biofilm formation can modify soil aggregation, ion mobility, and metal bioavailability, thereby altering the stress environment experienced by plant roots [9].

Climate change further complicates these interactions. Rising temperatures, shifting precipitation patterns, and more frequent extreme climate events affect plant physiology as well as microbial community composition and stability [10]. Under drought, for example, shifts in microbial carbon metabolism and phytohormone production may influence whether plants successfully acclimate [11]. Likewise, warming and soil acidification can alter metal speciation and microbial detoxification processes, with consequences for both crop performance and phytoremediation outcomes [12].

In this context, the MDPI Topic **“Microbe-Induced Abiotic Stress Alleviation in Plants”** provides a platform for integrating insights from molecular biology, microbial ecology, plant physiology, and soil science. The contributions provide diverse lines of evidence, although the research does not yet converge on a single, coherent framework.

## 2. Evidence Emerging from the Topic “Microbe-Induced Abiotic Stress Alleviation in Plants”

The contributions to this Topic highlight the multidimensional nature of microbial stress mitigation. Rather than pointing to a single dominant mechanism, the studies collectively suggest that multiple microbial functions operate across different stress contexts. Traits such as EPS production, phytohormone modulation (e.g., indole-3-acetic acid), metal chelation, and volatile organic compound (VOC) emission have been implicated in alleviating osmotic imbalance, oxidative damage, and ion toxicity in plants [13,14,15]. However, their effectiveness appears to be variable and often depends on environmental conditions and host–microbe compatibility. This variability is particularly evident in the case of microbial VOCs. Unlike root-associated interactions, VOC-mediated effects can occur without direct physical contact, suggesting a more diffuse mode of influence on plant physiology [14]. The reproducibility of these VOC-mediated effects remains uncertain under field conditions. More broadly, a synthesis study indicates that microorganisms can enhance plant tolerance to drought and salinity by influencing stress signaling pathways, antioxidant systems, and metabolic processes [16]. In cadmium (Cd)-contaminated systems, inoculation with EPS-producing, Cd-tolerant bacterium enhances plant antioxidant activity and growth while reducing Cd bioavailability through adsorption, chelation, and mineral precipitation. These effects often coincide with shifts in soil aggregation and rhizosphere communities, potentially limiting Cd uptake and increasing phytoremediation efficiency [17].

Evidence from this Topic suggests that microbial consortia can, in some cases, outperform single inoculants, particularly when complementary functional traits are involved. Interactions between plant growth–promoting rhizobacteria and arbuscular mycorrhizal fungi, for example, may enable the coordinated regulation of nutrient acquisition, stress signaling, and antioxidant responses [18]. However, these synergistic effects are not universal and often depend on environmental conditions, host specificity, and the stability of microbial interactions over time.

This context dependency becomes more apparent when considering broader biological interactions. Studies on plant viral coinfections show that outcomes can shift between antagonism and synergism depending on host tissue and physiological state, effectively and highly dynamically reshaping metabolic and defense responses [19]. Likewise, emerging metagenomic evidence indicates that aboveground endophytic fungi indirectly restructure rhizosphere microbial communities by altering functional gene abundance linked to carbon fixation and nitrogen cycling [20]. Such findings reinforce a conceptual shift from inoculating individual “beneficial strains” toward designing stress responsive microbial consortia capable of sustained functional stability across heterogeneous environments.

A further set of contributions links microbial activity to soil structure, aggregation, and biogeochemical cycling, demonstrating that microorganisms modify not only plant physiology but also the physical and chemical milieu in which roots function. By regulating soil aggregation, pore architecture, and mineral–organic interfaces, microbes modulate water retention, ion transport, and metal bioavailability. These soil-mediated pathways are particularly critical under salinity and heavy metal stress, where the spatial distribution and chemical speciation of ions ultimately determine toxicity and plant exposure. For instance, inoculation with *Enterobacter* sp. significantly increases plant biomass, Cd accumulation, and translocation in *Hybrid Pennisetum*, while reconfiguring the rhizosphere microbial community structure, increasing soil nutrient availability, and reshaping metabolic profiles, ultimately enhancing phytoremediation efficiency [21]. Metagenomic analyses indicate that the presence of symbiotic *Epichloë* endophytic fungi alters the functional potential of the rhizosphere microbiome by increasing the abundance of microbial genes associated with the reductive tricarboxylic acid carbon fixation pathway and nitrification, while promoting the accumulation of genes conferring resistance to heavy metals in the rhizosphere [20]. Complementary studies on plant–pathogen interactions reveal that biotic stress profoundly disrupts the photosynthetic machinery and cellular redox balance, as multiple viral infections suppress the expression of photosynthesis-related gene while simultaneously inducing antioxidant defenses, thereby reshaping plant metabolic homeostasis under stress [22]. These findings illustrate how microbial processes operating from molecular and physiological responses to microbial community dynamics and soil structural collectively shape plant resilience under environmental stress.

Rather than converging on a single narrative, these studies point to a more context-dependent picture: the effects of microbes on plant stress tolerance are shaped by soil properties, climate variability, and plant genotype. Interactions within microbial communities can either enhance or constrain these effects. Consistent improvements in plant performance will remain difficult to achieve unless these processes are collectively considered.

## 3. Future Perspectives: Predictive and Climate-Smart Plant–Microbiome Engineering

What, then, should define the next generation of research in microbe-induced stress alleviation?

The evidence assembled in this Topic points toward several priorities: (1) developing predictive models that integrate plant physiology, microbial community dynamics, and soil–climate interactions; (2) identifying keystone taxa and functional guilds that govern resilience under multiple-stress conditions; (3) linking microbial metabolites and signaling molecules to plant stress response pathways; (4) scaling up from controlled experiments to long-term, multisite field trials; (5) embedding microbial strategies within One Health and climate-smart agriculture frameworks.

Ultimately, the future of sustainable crop production under climate change will depend on our ability to harness the adaptive capacity of plant-associated microbiomes. Microbes are not simply inputs to be applied but partners to be managed, designed, and integrated into agroecosystems. By advancing a systems-level understanding of plant–microbe–soil interactions, this Topic seeks to contribute to a new paradigm in which microbial processes become central to environmental resilience, food security, and ecological sustainability.
